# Elevated CO_2_-Mitigation of High Temperature Stress Associated with Maintenance of Positive Carbon Balance and Carbohydrate Accumulation in Kentucky Bluegrass

**DOI:** 10.1371/journal.pone.0089725

**Published:** 2014-03-24

**Authors:** Yali Song, Jingjin Yu, Bingru Huang

**Affiliations:** 1 College of Forestry, Beijing Forestry University, Beijing, China; 2 College of Agro-Grassland Science, Nanjing Agricultural University, Nanjing, China; 3 Department of Plant Biology and Pathology, Rutgers University, New Brunswick, New Jersey, United States of America; Tennessee State University, United States of America

## Abstract

Elevated CO_2_ concentration may promote plant growth while high temperature is inhibitory for C_3_ plant species. The interactive effects of elevated CO_2_ and high temperatures on C_3_ perennial grass growth and carbon metabolism are not well documented. Kentucky bluegrass (*Poa pratensis*) plants were exposed to two CO_2_ levels (400 and 800 μmol mol^−1^) and five temperatures (15/12, 20/17, 25/22, 30/27, 35/32°C, day/night) in growth chambers. Increasing temperatures to 25°C and above inhibited leaf photosynthetic rate (Pn) and shoot and root growth, but increased leaf respiration rate (R), leading to a negative carbon balance and a decline in soluble sugar content under ambient CO_2_. Elevated CO_2_ did not cause shift of optimal temperatures in Kentucky bluegrass, but promoted Pn, shoot and root growth under all levels of temperature (15, 20, 25, 30, and 35°C) and mitigated the adverse effects of severe high temperatures (30 and 35°C). Elevated CO_2_-mitigation of adverse effects of high temperatures on Kentucky bluegrass growth could be associated with the maintenance of a positive carbon balance and the accumulation of soluble sugars and total nonstructural carbohydrates through stimulation of Pn and suppression of R and respiratory organic acid metabolism.

## Introduction

High temperatures during summer months is a primary factor limiting the growth of C_3_ cool-season plant species as temperatures often exceed the optimal range of 10 to 24 °C for shoot and root growth during these months in many areas [1]. Elevated temperature is becoming an increasingly significant abiotic stress in the scenario of global climate change, as temperature is predicted to increase more than 5.8°C by the end of this century [2]. The rise in temperature has been associated with increasing atmospheric CO_2_; atmospheric CO_2_ concentration has increased by 100 μmol mol^−1^ since the beginning of the industrialized era and is predicted to continue rising at a rate of approximately 2 μmol mol^−1^ per year [2]. Extensive effort has been taken to examine effects of elevated CO_2_ on plant growth under optimal or non-stressful temperature conditions and most studied reported positive effects on plant growth in various plant species [3]–[11]. However, limited studies reported the combined effects of elevated CO_2_ and elevated temperatures on plant growth [12]–[13]. Elevated CO_2_ was found to mitigate the adverse effects of heat stress on photosynthesis, water use, and overall plant growth in different plant species [12], [14]–[15], including C_3_ perennial grass species [16]–[17]. Few studies reported elevated CO_2_ may increase the optimum temperature for plant growth [18].

The mechanisms underlying positive effects of elevated CO_2_ on plant growth under non-stressful temperatures have been well documented, including increases in photosynthesis, reduction in transpiration rate and stomatal conductance, suppression of dark respiration and photorespiration, as well as affect the accumulation of carbohydrates [4], [11], [13], [16]–[17], [19]–[26]. However little is known on how elevated CO_2_ may mitigate growth inhibition and physiological damages under different levels of high temperatures beyond the optimal ranges, particularly for cool-season grass species, which are sensitive to increasing temperatures. Photosynthesis and respiration are among the most sensitive metabolic processes to increasing temperatures [27]. Under non-stressful temperatures, cool-season plants maintain a positive carbon balance with photosynthetic rates typically being greater than respiration rates, which is critically important for maintaining active plant growth [28]–[29] and for increasing carbon sequestration [30]. Increasing temperatures not only inhibit photosynthetic rate but enhance respiration rate under ambient CO_2_ conditions, causing the decline in the availability of carbohydrates for energy supply as well as carbon skeletons to support plant growth [31]–[33]. Furthermore, how elevated CO_2_ may affect carbon balance and metabolite accumulation under different levels of temperature is not well documented and whether elevated CO_2_-mitigation of the negative effects of high temperatures is associated with the maintenance of carbon balance and the accumulation of carbon metabolites is unclear.

Cool-season perennial grass species, including Kentucky bluegrass, which are used as forage and turf grasses are particularly sensitive to increasing temperatures [27]. Increasing temperatures not only adversely affect plant growth but also the carbon sequestration potential of cool-season perennial grass species. Understanding the mechanisms of how elevated CO_2_ may affect response of cool-season grasses to increasing temperature is important for promoting growth and adaptation to increasing temperatures. The objectives of this study were to investigate 1) whether elevated CO_2_ may cause shift in the optimal temperatures for Kentucky bluegrass growth by examining shoot growth, and root growth, as well as photosynthetic responses to increasing temperatures (15, 20, 25, 30, and 35°C) under elevated CO_2_ or ambient CO_2_ conditions, and 2) to determine whether CO_2_-mitigation of adverse effects of high temperatures was associated with the maintenance of positive carbon balance and the accumulation of photosynthetic and respiratory metabolites.

## Materials and Methods

### Plant Materials and Growing Conditions

Kentucky bluegrass (cv. ‘Baron’) plants were collected from turfgrass field plots at the Rutgers University research farm in Adelphia, NJ. Plants were propagated in pots (10 cm in diameter, 40 cm in height) filled with fritted clay in a greenhouse with average day/night temperatures of 21/18°C (day/night) and 12 h natural light at 750 μmol m^−2^s^−1^ photosynthetically active radiation (PAR) for 38 d. During establishment, plants were trimmed weekly to maintain a canopy height at 10 cm, irrigated every two days, and fertilized every three days with half-strength Hoagland's nutrient solution [34]. Plants were then transferred to growth chambers (Environmental Growth Chamber, Chargrin Fall, Ohio, USA) for CO_2_ and temperature treatments.

### Treatments and Experimental Design

For the examination of CO_2_ effects, plants were exposed to two CO_2_ treatments: ambient CO_2_ (400±20 μmol mol^−1^) and elevated CO_2_ (800±20 μmol mol^−1^). Each CO_2_ treatment was replicated in four growth chambers, and each treatment was re-randomized between chambers during the treatment period to avoid confounding chamber effects. The concentration of CO_2_ inside each growth chamber was maintained through an automated, open-chamber CO_2_ control system connected to a gas tank containing 100% CO_2_ (Airgas, Inc.) using the design described in [16]. The different CO_2_ levels were continuously monitored through an infrared gas analyzer (Li-820, LICOR, Inc.) and controlled using an automatic system consisting of a programmable logic controller unit, solenoid valves, and a laptop computer with monitoring software accurate to within 20 μmol mol^−1^ of the target levels (400 and 800 μmol mol^−1^). Plants were maintained at the two CO_2_ levels for 2 weeks which allowed sufficient time for the formation of new leaves under CO_2_ treatments, prior to the exposure of plants to different temperature treatments.

Plants exposed to either ambient or elevated CO_2_ treatment were subjected to five temperatures: 15/12, 20/17, 25/22, 30/27, or 35/32 °C (day/night). The temperature treatments were conducted over time in sequential order, and each treatment was repeated in four growth chambers. Plants were relocated among the different chambers once per week to minimize confounding effects of environmental variation between different chambers. Other environmental conditions in the growth chamber were 70% relative humidity, 660 μmol m^−2^s^−1^ PAR, and a 12-h photoperiod. Plants were well-watered and fertilized as described above.

### Growth Analysis

Turf visual quality (TQ) was used as an indicator for overall turf performance and rated based on shoot density, uniformity, and color on a scale of 1 (lowest, completely desiccated and brown canopy) to 9 (best, fully turgid and green turf canopy) [35].

At the end of the experiment, shoots and roots were collected. Roots were washed free of fritted clay. Samples were dried in an oven at 82°C for 72 h and total shoot and root dry weight was determined. Root to shoot ratio (root/shoot) was calculated using root and shoot dry weight.

### Determination of Single Leaf Net Photosynthetic Rate (Pn) and Dark Respiration Rate (R)

Leaf photosynthesis (Pn) and leaf dark respiration (R) rates were measured once a week on six second and third fully-expanded leaves per replicate pot with a portable infrared gas exchange system (LI6400, LI-COR Inc., Lincoln, NE). Leaves were placed into a 2×3 cm standard leaf chamber containing a built-in red and blue LED light source and Pn measured at PAR 800 μmol photon m^−2^ s^−1^. Dark respiration rate was measured using the LI-6400 infrared gas analyzer with leaves enclosed in the chamber without light supply. For both Pn and R, the analyzer was set at 500 μmol s^−1^ flow rate and 70% relative humidity. CO_2_ level and temperature were set depending on the individual treatment of the plants as described prior.

### Carbohydrate and Organic Acid Analysis

Leaves and roots were lyophilized and subsequently ground to a fine powder using a mortar and pestle. The samples were stored in a −80°C freezer. Total non-structural carbohydrate (TNC) content was analyzed according to the method described by [36] with modifications. Fifty milligrams of ground samples were transferred to glass tubes containing 2.5 ml of 5.0% amylase and incubated at 37°C for 24 h. After 24 h, 0.5 ml of 0.6 N HCl was added to the solution and samples incubated for an additional 18 h. Following incubation, 0.31 ml of 10 N NaOH was added to adjust the pH of the solution to between 5 and 7. Solutions were transferred to round-bottom flasks, volume adjusted to 50 ml with deionized water and solutions filtered. A 1.0 ml aliquot of solution was transferred to a glass tube containing 1.5 ml alkaline ferricyanide solution. Solution was then placed in a boiling water bath for 10 min, quickly cooled in an ice bath, and then partially neutralized with 3.0 ml of 2 N H_2_SO_4_. Finally, 1.2 ml arsenomolybdate solution was added and the total volume adjusted to 25 ml with deionized water. The absorbance of the solution was measured at 515 nm with a spectrophotometer (Spectronic Genesys Series; Spectronic Instruments, Rochester, N.Y.) and TNC content calculated using a standard curve.

Soluble sugars and organic acids were analyzed using GC/MS according to the procedure described in [37] and modified from [38]. The derived extracts were analyzed with a PerkinElmer gas chromatograph coupled with a TurboMass- Autosystem XL mass spectrometer (Perkin Elmer Inc., Waltham, MS). A 1 μl extract aliquot of the extracts was injected into a DB-5MS capillary column (30 m×0.25 mm×0.25 μm) (Agilent J&W Scientific, Folsom, CA). The inlet temperature was set at 260 °C. After a 5-min solvent delay, initial GC oven temperature was set at 80°C; 2 min after injection, the GC oven temperature was raised to 280°C with 5°C min^−1^, and finally held at 280°C for 13 min. The injection temperature was set to 280°C and the ion source temperature was adjusted to 200°C. Helium was used as the carrier gas with a constant flow rate set at 1 ml min^−1^. The measurements were made with electron impact ionization (70 eV) in the full scan mode (m/z 30–550). The metabolites were identified using TURBOMASS 4.1.1 Software (Perkin Elmer Inc.) coupled with commercially available compound libraries: NIST 2005(Perkin Elmer Inc.,Waltham, MS),Wiley 7.0 (John Wiley & Sons Ltd., Hoboken, NJ).

### Statistical Analysis

Main treatment effects and interactive effects of CO_2_ and temperature were determined by analysis of variance (ANOVA) according to the general linear model procedure of SAS (SAS 9.1; SAS Institute Inc., Cary, NC). Differences between means were separated by Fisher's protected least significance difference (LSD) test at the 0.05 probability level.

## Results

### Turf Quality, Shoot and Root Biomass

**Figure 1:**
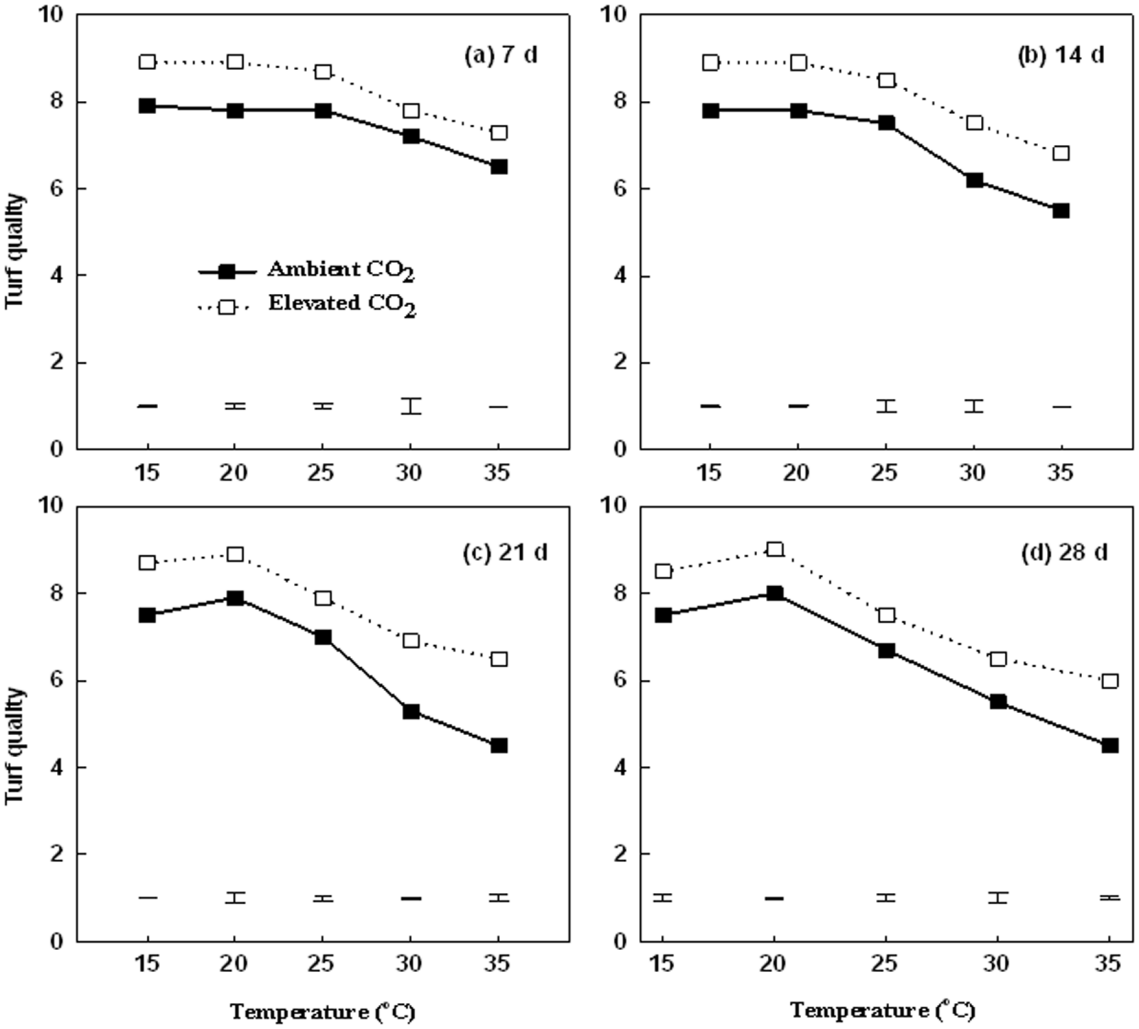
Effects of elevated CO_2_ on turf quality responses to increasing temperatures at 7 d (a), 14 d (b), 21 d (c), and 28 d (d) of temperature treatment. Vertical bars represent the values of least significant difference at p = 0.05 for comparison of CO_2_ treatment effects at a given temperature. The LSD value for comparisons between temperature treatments was 0.1198 and 0.0284 under ambient and elevated CO_2_ concentration, respectively, at 7 d, 0.0283 and 0.0229 at 14 d, 0.1909 and 0.1685 at 21 d, and 0.1928 and 0.1732 at 28 d.

Under both ambient and elevated CO_2_ levels, turf quality remained unchanged from 15 to 25°C, but declined as temperature increased to 30 and 35°C after 14 d of treatment, and this decline became more pronounced with prolonged periods of treatment at 21 and 28 d (Fig. 1). TQ of plants exposed to ambient CO_2_ decreased below the minimum acceptable value (6.0) at 14 d of 35°C and at 21 and 28 d of in both 30 and 35°C, whereas that of plants exposed to elevated CO_2_ did not drop below 6.0 at any temperatures at any day of treatment. Elevated CO_2_ did not raise the temperature at which TQ decline first occurred, but significantly increased TQ across all temperature treatments.

**Figure 2:**
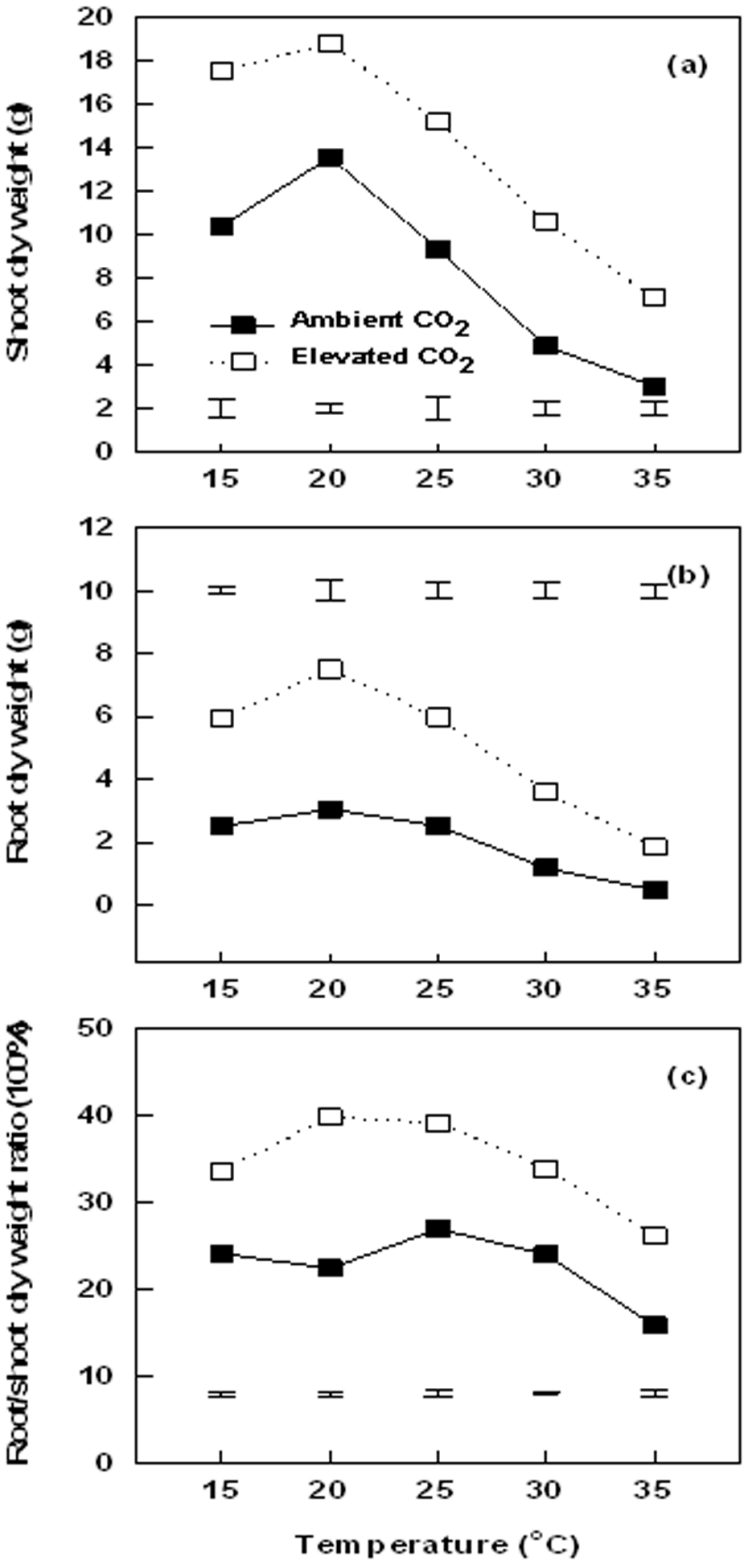
Effects of elevated CO_2_ on the responses of shoot dry weight (a), root dry weight (b), and root/shoot dry weight ratio (c) to increasing temperatures at 28 d of temperature treatment. Vertical bars represent the values of least significant difference at p = 0.05 for comparison of CO_2_ treatment effects at a given temperature. The LSD value for comparisons between temperature treatments was 0.7078 and 0.9462 under ambient and elevated CO_2_ concentration, respectively.

Total shoot biomass (Fig. 2a) and root biomass (Fig. 2b) were highest at 20°C, and decreased to the lowest level with increasing temperatures up to 35°C under both ambient and elevated CO_2_ conditions. Root/shoot ratio remained constant from 15 to 30°C, and then decreased at 35°C under both ambient and elevated CO_2_ conditions (Fig. 2c). Elevated CO_2_ increased both shoot and root biomass under all levels of temperature treatment. Compared to ambient CO_2_ treatments, elevated CO_2_ resulted in significantly higher root/shoot ratio across all temperature treatments.

### Leaf Net Photosynthetic Rate (Pn) and Dark Respiration Rate (R)

**Figure 3:**
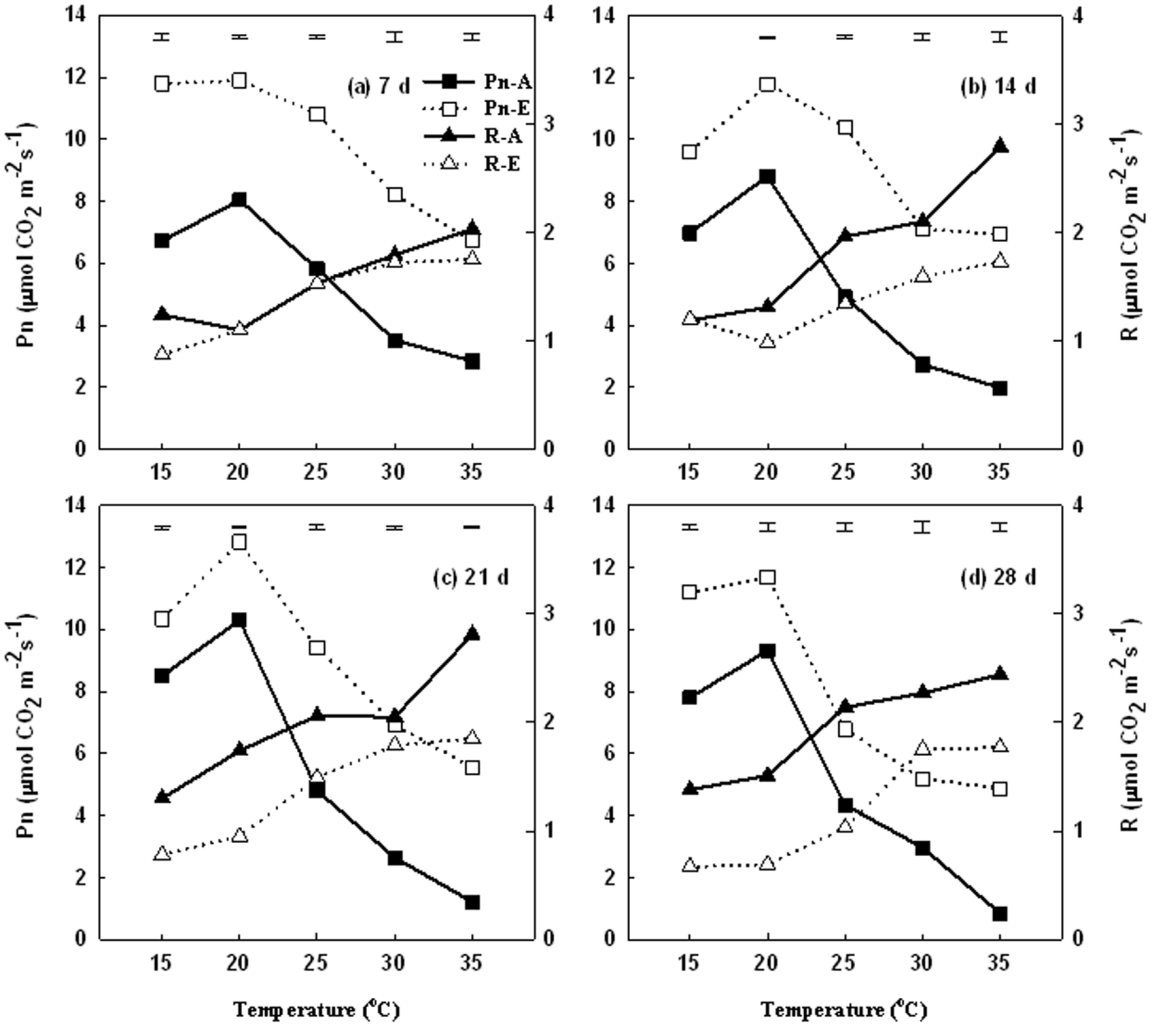
Effects of elevated CO_2_ on the responses of leaf photosynthetic rate (Pn) and leaf respiration rate (R) to increasing temperatures at 7 d (a), 14 d (b), 21 d (c), and 28 d (d) of temperature treatment, primary vertical axis for Pn, secondary vertical axis for R. Vertical bars represent the values of least significant difference at p = 0.05 for comparison of CO2 treatment effects at a given temperature. The LSD value for comparisons between temperature treatments was 0.2885 and 0.3068 under ambient and elevated CO_2_ concentration, respectively, at 7 d, 0.3812 and 0.2818 at 14 d, 0.5131 and 0.3814 at 21 d, and 0.4642 and 0.4347 at 28 d.

Leaf photosynthetic rate (Pn) was the highest at 20°C and decreased with increasing temperatures at 25, 30, and 35°C under either ambient or elevated CO_2_ (Fig. 3). Plants at 35°C had the lowest Pn during the entire treatment period (28 d); this was observed under both ambient and elevated CO_2_ conditions. The reduction in Pn when comparing 20 to 35°C was 65, 77, 88, and 91% at 7, 14, 21, and 28 d of treatment, respectively, under ambient CO_2_; the coresponding percent reductions were 43, 41, 57, and 58% at 7, 14, 21, and 28 d of treatment under elevated CO_2_.

Elevated CO_2_ resulted in significantly higher Pn at all temperatures at 7, 14, 21, and 28 d of treatment (Fig. 3), particularly under higher temperatures (25, 30, and 35°C) after 14 d of treatment (Fig. 3b), when compared to the ambient CO_2_ level. Elevated CO_2_ increased Pn by 89, 130, and 330% at 25, 30, and 35°C, respectively, averaged over the data at 14, 21, and 28 d of treatment (Fig. 3b,c,d).

Leaf respiration rate (R) increased with increasing temperatures from 15 to 35°C under both ambient and elevated CO_2_ (Fig. 3). Elevated CO_2_ did not have significant effects on leaf R under any temperature levels at 7 d of treatment (Fig. 3a), but suppressed leaf R from 20 to 35°C at 14 d (Fig. 3b) and under all temperature levels at 21 d (Fig. 3c) and 28 d (Fig. 3d). Elevated CO_2_ lead to the reduction in leaf R by 32, 43, 37, 20, and 33% at 15, 20, 25, 30, and 35°C, respectively, averaged over the data at 14, 21, and 28 d of treatment.

**Figure 4:**
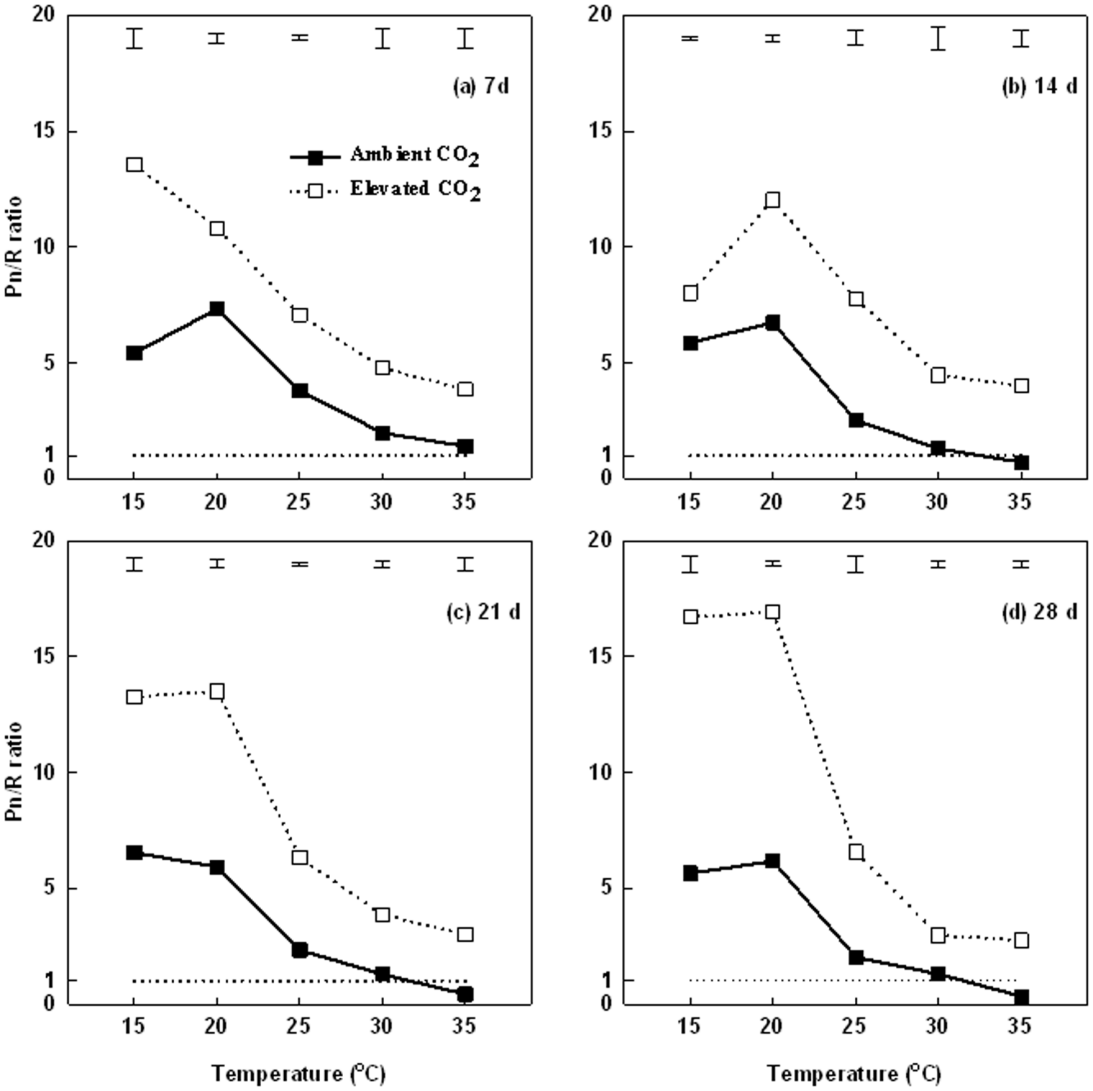
Effects of elevated CO_2_ on leaf photosynthetic rate (Pn) to leaf respiration rate (R) ratio (Pn/R) at different temperatures at 7 d (a), 14 d (b), 21 d (c), and 28 d (d) of temperature treatment, and the dotted line represents Pn/R ratio was 1.0. Vertical bars represent the values of least significant difference at p = 0.05 for comparison of CO_2_ treatment effects at a given temperature.

The Pn/R ratios was greatest at 15 and 20°C, and decreased with increasing temperatures to 25, 30, and 35°C during the entire treatment period under both ambient and elevated CO_2_ treatments (Fig. 4). Under ambient CO_2_, the ratio decreased to close to 1.0 at 7 d of 35°C (Fig. 4a) and at 14 d (Fig. 4b) and 21 d (Fig. 4c) of 30°C; Pn/R ratio decreased to below 1.0 at 35°C at both 21 (Fig. 4c) and 28 d (Fig. 4d). Under elevated CO_2_, the Pn/R ratio was maintained above 1.0 and was significantly greater than that under ambient CO_2_ under all temperature levels.

### Carbohydrate and Organic Acid Accumulation

**Figure 5:**
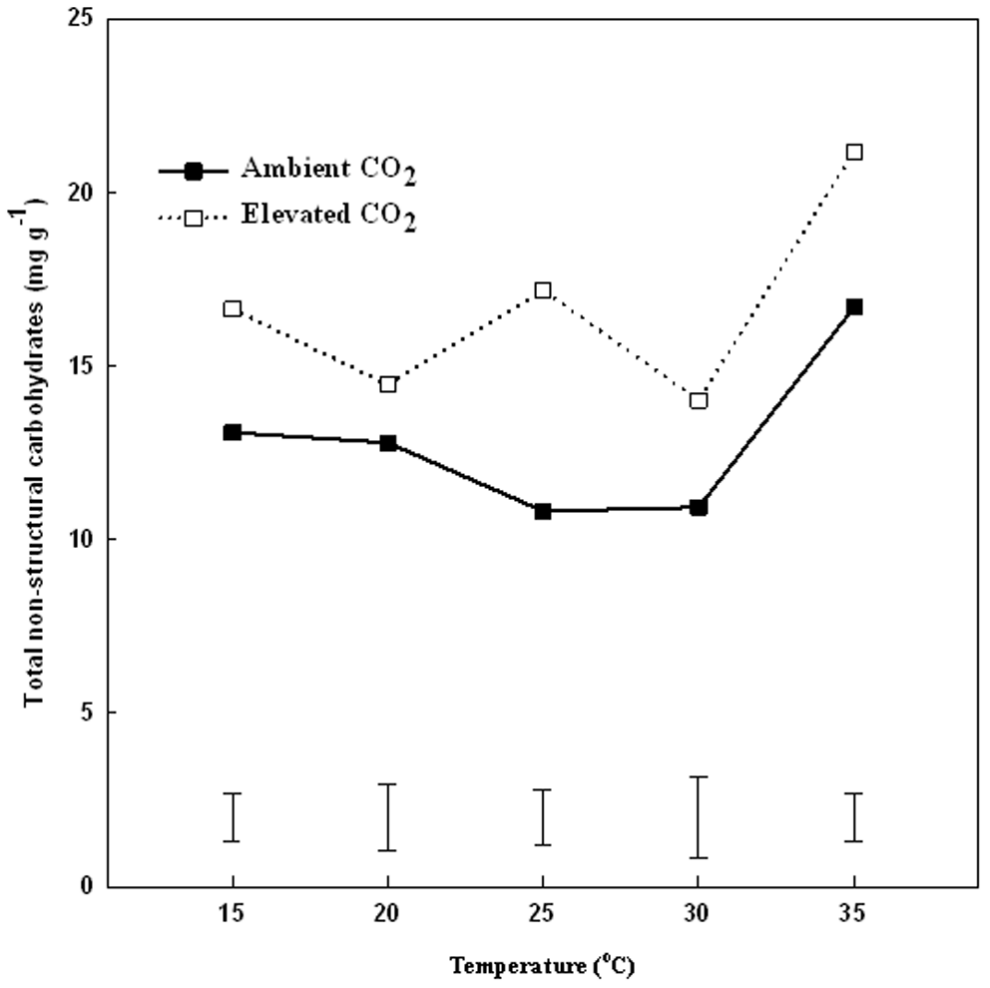
Effects of elevated CO_2_ on total non-structural carbohydrates in leaves at 28 d of different temperature treatments. Vertical bars represent the values of least significant difference at p = 0.05 for comparison of CO_2_ treatment effects at a given temperature. The LSD value for comparisons between temperature treatments was 0.3392 and 0.3993 under ambient and elevated CO_2_ concentration, respectively.

Total non-structural carbohydrate (TNC) content in leaves was not significantly different at temperatures 20 and 30°C, but increased by 30% and 46% at 35°C under ambient and elevated CO_2_, respectively, compared to that at the lower temperatures (Fig. 5). Under elevated CO_2_, TNC content was significantly higher (by 38%) than that under ambient CO_2_ at 25–35°C (Fig. 5).

**Figure 6:**
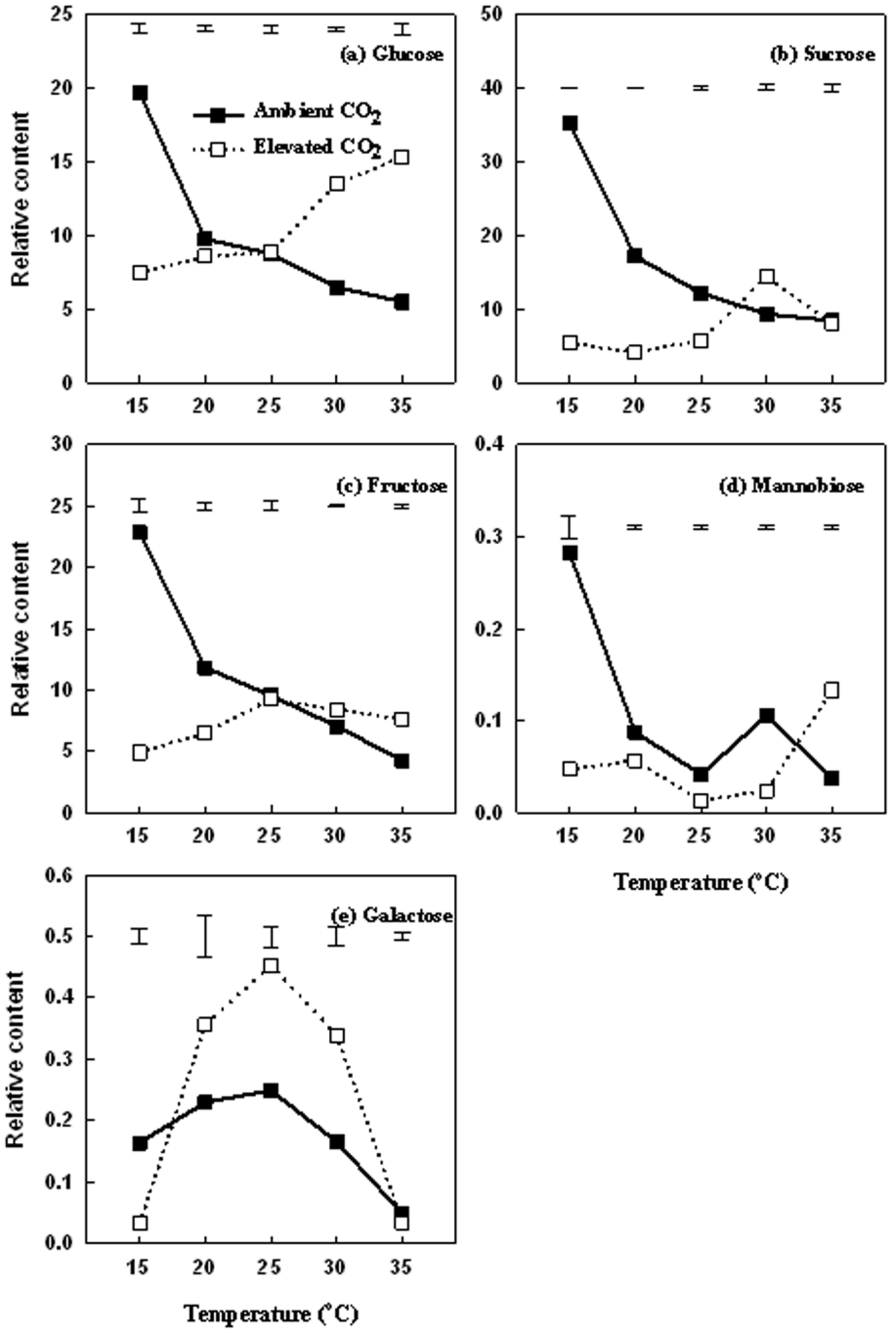
Effects of elevated CO_2_ on the relative soluble sugars content in leaves at 28 d of different temperature treatments. Vertical bars represent the values of least significant difference at p = 0.05 for comparison of CO_2_ treatment effects at a given temperature. The LSD value for comparisons between temperature treatments was 0.0183 and 0.0223 under ambient and elevated CO_2_ concentration, respectively.

The content of soluble sugars in leaves, including glucose (Fig. 6a), sucrose (Fig. 6b), fructose (Fig. 6c), and mannobiose (Fig. 6d), exhibited significant decline with increasing temperatures from 15 to 35°C under ambient CO_2_. Under elevated CO_2_, glucose content increased with increasing temperatures (Fig. 6a); sucrose content was highest at 30°C but declined thereafter (Fig. 6b); fructose content did not change with increasing temperatures (Fig. 6c); mannobiose content did not change between 15 and 30°C, but increased at 35°C. The content of galactose increased as temperature increased from 15 to 25 and then decreased at 30 and 35°C under both ambient and elevated CO_2_ (Fig. 6e). Elevated CO_2_ resulted in a significantly higher content of glucose (by 144%) at 30 and 35°C (Fig. 6a), sucrose content (by 55%) at 30°C (Fig. 6b), fructose content (by 80%) (Fig. 6c) and mannobiose content (by 254%) (Fig. 6d) at 35°C, and galactose content (by 80%) at 20, 25, and 30°C (Fig. 6e), when compared to ambient CO_2_ conditions.

**Figure 7:**
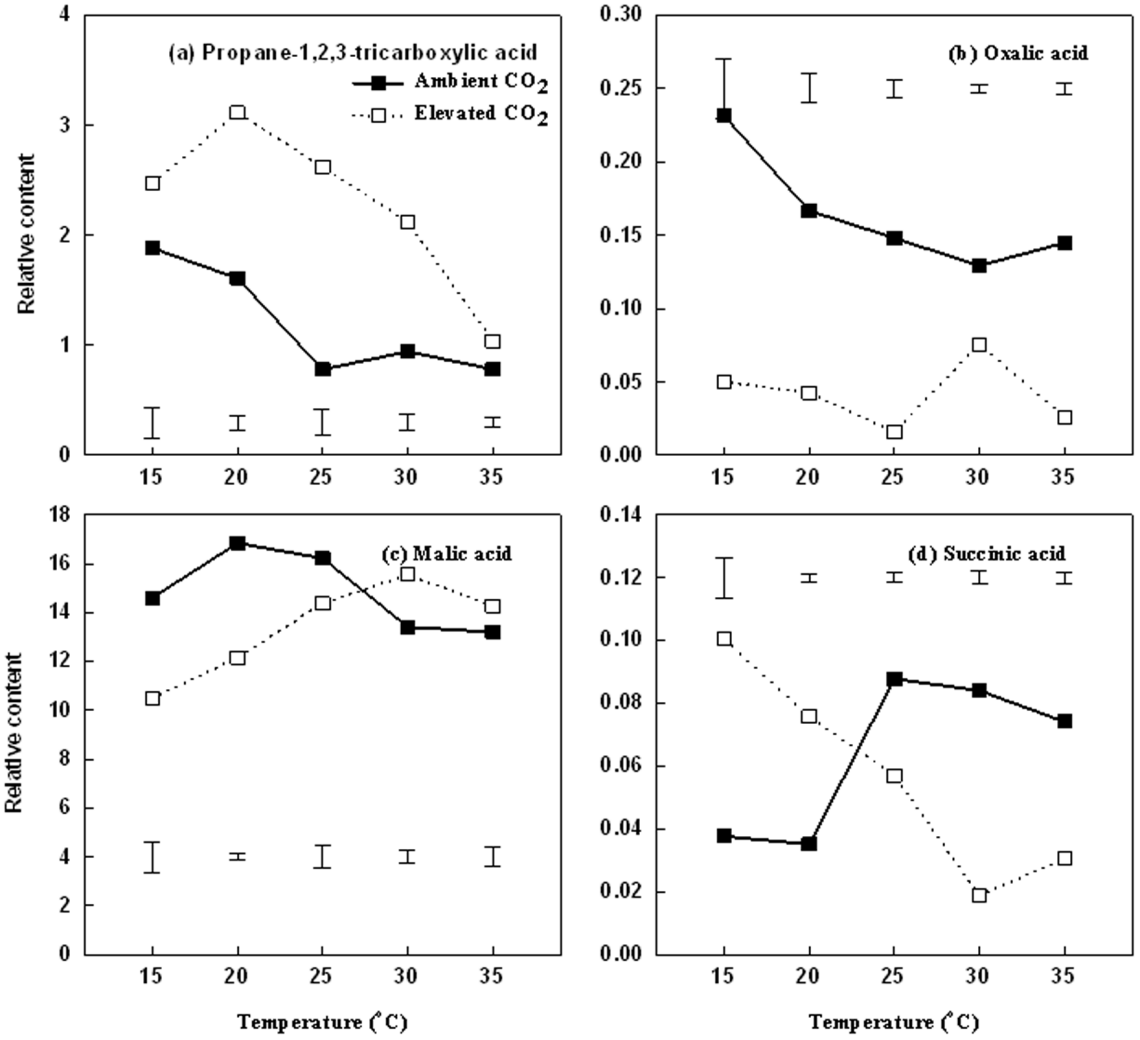
Effects of elevated CO_2_ on the relative organic acids content in leaves at 28 d of different temperature treatments. Vertical bars represent the values of least significant difference at p = 0.05 for comparison of CO_2_ treatment effects at a given temperature. The LSD value for comparisons between temperature treatments was 0.0318 and 0.0171 under ambient and elevated CO_2_ concentration, respectively.

The content of several major organic acids involved in respiratory metabolism exhibited differential responses to increasing temperatures and CO_2_ treatments (Fig. 7). Under ambient CO_2_, propane-1, 2, 3-tricarboxylic acid (PTC) (Fig. 7a) and oxalic acid (Fig. 7b) decreased from 15 to 25°C and became steady at 30 and 35°C. Malic acid content also had a decrease under ambient CO_2_ conditions from 25 to 35°C (Fig. 7c); the content of succinic acid increased with increasing temperatures from 15 to 25°C and decreased thereafter with higher temperature (Fig. 7d). Under elevated CO_2_, PTC content was the highest at 20°C and then decreased from 25 to 35°C (Fig. 7a); oxalic acid content did not change significantly with increasing temperatures (Fig. 7b); malic acid content increased with temperature to the highest level at 30 and then decreased at 35°C (Fig. 7c); succinic acid content exhibited decline from 15°C to 30°C (Fig. 7d).

Elevated CO_2_ resulted in significantly higher PTC content under all levels of temperature compared to the ambient CO_2_ treatment (Fig. 7a), but caused significant reduction in oxalic acid content under all temperature levels (Fig. 7b). Malic acid content of plants exposed to elevated CO_2_ was significantly lower at 15–25°C and higher at 30°C, but was not significantly different from those plants treated with ambient CO_2_ at 35°C (Fig. 7c). Succinic acid content of plants exposed to elevated CO_2_ was significantly higher at 15 and 20°C, but significantly lower than that under ambient CO_2_ at 25, 30 and 35°C (Fig. 7d).

## Discussion

Kentucky bluegrass maintained highest turf quality levels and single leaf Pn at 15 and 20°C and highest shoot and root biomass at 20°C. Temperatures above 25°C resulted in declines in all these parameters under both ambient and elevated CO_2_ conditions. These results demonstrated that temperatures above 25°C were detrimental for Kentucky bluegrass growth and photosynthetic activities. It was reported that the range of optimal temperature requirements is between 10–24°C for cool-season grass species [1], [39]. Superaoptimal temperatures are detrimental for cool-season grass growth [27]. Root growth of Kentucky bluegrass was more sensitive to high temperatures than shoot growth, as reflected by lowering root/shoot ratio with increasing temperatures. Higher sensitivity of root growth than shoot growth in response to high temperatures has also been reported in other grass species [27], [40]–[42].

Few studies have shown effects of elevated CO_2_ on the shift of temperature optimum for photosynthesis or shoot and root growth. Using the non-rectangular hyperbola model of photosynthetic responses to increasing temperatures, Cannell and Thornley [18] predicted that elevated CO_2_ could raise the optimum temperature for leaf or canopy photosynthesis for plants acclimated to high temperature and high irradiance conditions. An interesting finding in this study was that elevated CO_2_ did not cause shifts in the upper range of the optimum temperatures for photosynthesis, shoot and root growth and in Kentucky bluegrass, which is a cool-season grass with 20°C being the optimum temperature under either CO_2_ conditions. However, elevated CO_2_ enhanced shoot and root growth, as well as photosynthetic activities under the different temperature levels, particularly under severely high temperatures (30 and 35°C) when compared to plants under ambient CO_2_ treatment. Our results suggest that elevated CO_2_ may alter the magnitude of the response of growth and photosynthetic activities to increasing temperatures without altering the temperature optimum for cool-season grass species.

Increases in shoot and root biomass by elevated CO_2_ under non-stress conditions have been reported in various other plant species [23], [43], [44], but few studies examined the differential responses of shoots and roots to elevated CO_2_ under different levels of temperature stress. In this study, the root/shoot biomass ratio also increased under elevated CO_2_ at temperatures from 15 to 35°C, particularly at 35°C, which could be the result of elevated-CO_2_ causing a greater increase in root growth than shoot growth for Kentucky bluegrass. Biomass allocation patterns between shoots and roots are a key determinant of plant growth, particularly for stress adaptation [23], [45]–[47]. More enhanced root growth relative to shoot growth by elevated CO_2_ could facilitate water and nutrient uptake by the root system to support plant growth and survival under high temperature stress.

High temperature suppression of shoot growth (lower turf quality and shoot biomass) and root growth (lower root biomass) could be related to the imbalanced photosynthesis and respiration. Increases in respiration rate in response to high temperature have been reported in various plant species [16], [48]–[55], including Kentucky bluegrass [56]. Respiration is a major avenue of carbon loss for plants and increased respiration rate can cause carbohydrate depletion especially with increasing temperatures [57]. Under ambient CO_2_ leaf Pn was greater than leaf R at temperatures below 25°C, but R exceeded Pn at 30 and 35°C by the end of the treatment period (28 d), which lead to the decreased ratio of Pn/R. Xu and Huang [40] reported that canopy respiration rate exceeded canopy Pn as temperature increased to 30°C in creeping bentgrass (*Agrostis stolonifera.*). Carbon is in the balance status when Pn and R are equal, but a Pn/R ratio less than 1.0 indicates that carbon consumption rate exceeds carbon production rate in photosynthesizing organs, which can lead to carbohydrate depletion and growth suppression. Positive carbon balance and carbohydrate accumulation is particularly important for maintaining shoot and root growth, as well as plant survival of higher temperatures, but the imbalanced carbon relation can be detrimental for plant adaptation to heat stress [40]. The Pn/R ratio decreased with increasing temperatures to below 1.0 following prolonged periods of high temperatures at 30 and 35°C, indicating that carbohydrate consumption exceeded carbohydrate production under high temperatures, which could lead to the decline in carbohydrate availability or carbohydrate depletion. The lower Pn/R ratio with increasing temperature indicated a negative carbon balance, which was associated with the decline in photosynthetic rate and an increase in respiration rate during both ambient and elevated CO_2_. The content of soluble sugars, including glucose, sucrose, fructose, mannobiose, and galactose, which are assimilates from photosynthesis, indeed exhibited significant decline with increasing temperatures The imbalanced Pn and R under high temperatures could play parts in the decline in the availability of carbohydrates, particularly soluble sugars, which can limit shoot and root growth, although other metabolic factors, such as the inhibition of the capacity to convert starch to soluble sugars could also be involved.

Elevated CO_2_ promoted leaf Pn by an average of 109% across different levels of temperature. Increases in Pn under elevated CO_2_ have been reported in other plant species. For example, among grass species the increases in Pn enhanced by elevated CO_2_ concentrations relative to ambient CO_2_ (400 ppm) varied from 15% in 13 prairie grassland species [25] with 560 ppm CO_2_ to 162% in tall fescue with 800 ppm CO_2_ [16], and others in between with a 30% increases in ryegrass at 700 ppm CO_2_ [58], 65% increase at 510 ppm in *Deschampsia flexuosa* [59]. Elevated CO_2_-enhanced Pn has been associated with increases in carboxylation by Rubisco and decreased stomatal opening, regeneration capacity of ribulose-1,5-bisphosphate (RuBP), and suppression of photorespiration [18], [23], [60]–[65]. The enhanced Pn by elevated CO_2_ in Kentucky bluegrass could be related to changes in these metabolic activities, although they were not measured in this study, but are worth further investigation in future studies.

Many studies reported a decrease in respiration under elevated CO_2_ [16], [20], [66], [67], while some others found increases in respiration rate [66], [68] or no changes in respiration rate in response to elevated CO_2_ [16], [20]. The reasons for the discrepancy in respiration responses to elevated CO_2_ are still in debate, but the measuring system of respiration (CO_2_ evolution rate or O_2_ uptake rate), basis of respiration rate calculation (leaf area or leaf biomass), CO_2_ concentration, variable plant species (i.e. C_3_ vs. C_4_ plants) and environmental conditions in different studies may contribute to the contrasting effects of elevated CO_2_ on respiration rate [20], [66]. Leakey et al. (2009) pointed out that the inconsistent information on respiration was primarily due to lack of understanding of mechanisms controlling respiration responses to elevated CO_2_. In a review article, it is reported an approximately 20% reduction of respiration in leaves and roots in various plant species for doubling atmospheric CO_2_ [67]. Plants tend to become more efficient in carbon usage under elevated CO_2_ [26], [69]. In this study, elevated CO_2_ suppressed leaf R by an average of 28% across different levels of temperature. The effects of elevated CO_2_ on respiration rate have been associated with the direct inhibitory effects on mitochondrial electron transport enzymes, cytochrome c oxidase and succinate dehydrogenase during short-term exposure to elevated CO_2_ [57], [67] and the reduction in tissue nitrogen content and increase in soluble carbohydrates in plants exposed to long-term elevated CO_2_ treatment [48], [70]. In this study, the content of several major organic acids (oxalic acid, citric acid, and succinic acid) in the tricarboxylic acid cycle of respiration decreased under elevated CO_2_, particularly at high temperatures above 25°C, reflecting the suppression of respiratory activities by elevated CO_2_. Propane-1,2,3-tricarboxylic acid is an inhibitor of aconitase involved in carbon oxidation in the TCA cycle [71]. The content of PTC increased under elevated CO_2_, suggesting that PTC accumulation under elevated CO_2_ may interfere with the TCA cycle and be involved in the suppression of respiration.

A few studies examined the interactive effects of elevated CO_2_ and increasing temperatures on the carbon balance between photosynthesis and respiration [72]. Elevated CO_2_ facilitated the maintenance of Pn/R ratio above 1.0 at all temperature levels in the current study. The positive carbon gain under elevated CO_2_ was reflected by the higher content of TNC and soluble sugars in plants. Hunt et al. [73] illustrated that elevated CO_2_ increased TNC in prairie grasses. Casella and Soussana [58] reported elevated CO_2_ increased leaf fructan concentration by 189% in perennial ryegrass swards. As discussed earlier, elevated CO_2_ could be effective in the mitigation of more severe heat stress by enhancing photosynthetic production of carbohydrates and suppression of respiratory consumption of carbohydrates.

In summary, elevated CO_2_ concentration did not cause a shift in the optimal temperature level for shoot and root growth, as well as photosynthetic rate, but promoted these activities under all levels of temperature (15, 20, 25, 30, and 35°C) for Kentucky bluegrass. In addition, elevated CO_2_ mitigated the adverse effects of severely high temperatures (30 and 35°C). The promotive effects of elevated CO_2_ on Kentucky bluegrass growth could be attributed by the maintenance of positive carbon balance by stimulating leaf net photosynthetic rate and suppressing respiration rate, leading to the accumulation of soluble sugars and total nonstructural carbohydrates. Elevated CO_2_ could potentially increase the adaptability of these species to increasing temperatures by affecting carbon metabolism.
